# A rare case of multiple brain abscesses caused by apical periodontitis of deciduous teeth in congenital heart disease: a case report

**DOI:** 10.1186/s12903-022-02294-z

**Published:** 2022-06-28

**Authors:** Shizuka Takahashi, Hisato Segoe, Takashi Kikuiri, Yuji Maruo, Tomonobu Sato, Yutaka Watanabe, Zhao Jimei, Yoshitaka Yoshimura, Misa Ishiyama, Atsuhito Takeda, Yasutaka Yawaka, Tetsuo Shirakawa

**Affiliations:** 1 Department of Dentistry for Children and Disabled Person, Graduate School of Dental Medicine, Hokkaido University, Sapporo, 060-8586 Japan; 2 Department of Pediatrics, Japanese Red Cross Kitami Hospital, Kitami, 090-8666 Japan; 3 Department of Pediatric Dentistry, Nihon University School of Dentistry, Tokyo, 101-8310 Japan; 4 Department of Dentistry for Gerodontology, Dental Medicine and Graduate School of Dental Medicine, Hokkaido University, Sapporo, 060-8586 Japan; 5 Department of Molecular Cell Pharmacology, Graduate School of Dental Medicine, Hokkaido University, Sapporo, 060-8586 Japan; 6 Department of Pediatrics, Graduate School of Medicine, Hokkaido University, Sapporo, 060-8648 Japan

**Keywords:** Multiple brain abscesses, Apical periodontitis, Deciduous teeth caries, Congenital heart disease

## Abstract

**Background:**

A brain abscess is a focal infection in which abscesses form in the brain. A brain abscess is a rare but fatal disease when rupture occurs into the ventricles. We report a case of multiple brain abscesses caused by a hematogenous infection from the apical periodontitis of deciduous teeth.

**Case presentation:**

The patient was a 7-years and 8-months-old male with congenital heart disease. The patient sought medical attention due to fever and headache, for which he was started on three antibiotics with a diagnosis of multiple brain abscesses. Given that apical periodontitis of deciduous teeth was strongly suspected as the source of the brain abscess, the deciduous teeth were extracted. Immediately after deciduous teeth extraction, the patient’s headache and neurological symptoms disappeared.

**Conclusions:**

After teeth extraction, a clear shrinkage of the brain abscess was observed, and the patient was discharged from the hospital.

## Background

Mandibular osteomyelitis originating from dental infections occurs quite commonly. Although appropriate anti-inflammation and causative teeth treatment can treat this disease, inappropriate treatment can lead to a serious condition that places the patient’s life at risk. Brain abscesses are clinically serious infections of the central nervous system, characterized primarily by purulent bacteria that cause focal accumulation of pus in the brain parenchyma [1, 2].

While the infection routes of brain abscesses include direct transmission from proximate infectious foci such as otitis media and sinusitis, direct infection during open head trauma and brain surgery, and hematogenous infection from respiratory infections and endocarditis, such a condition is rarely caused by dental infections [3–5]. We herein report a case of multiple brain abscesses that could have resulted from dental infections of deciduous teeth.

## Case presentation

**Fig. 1:**
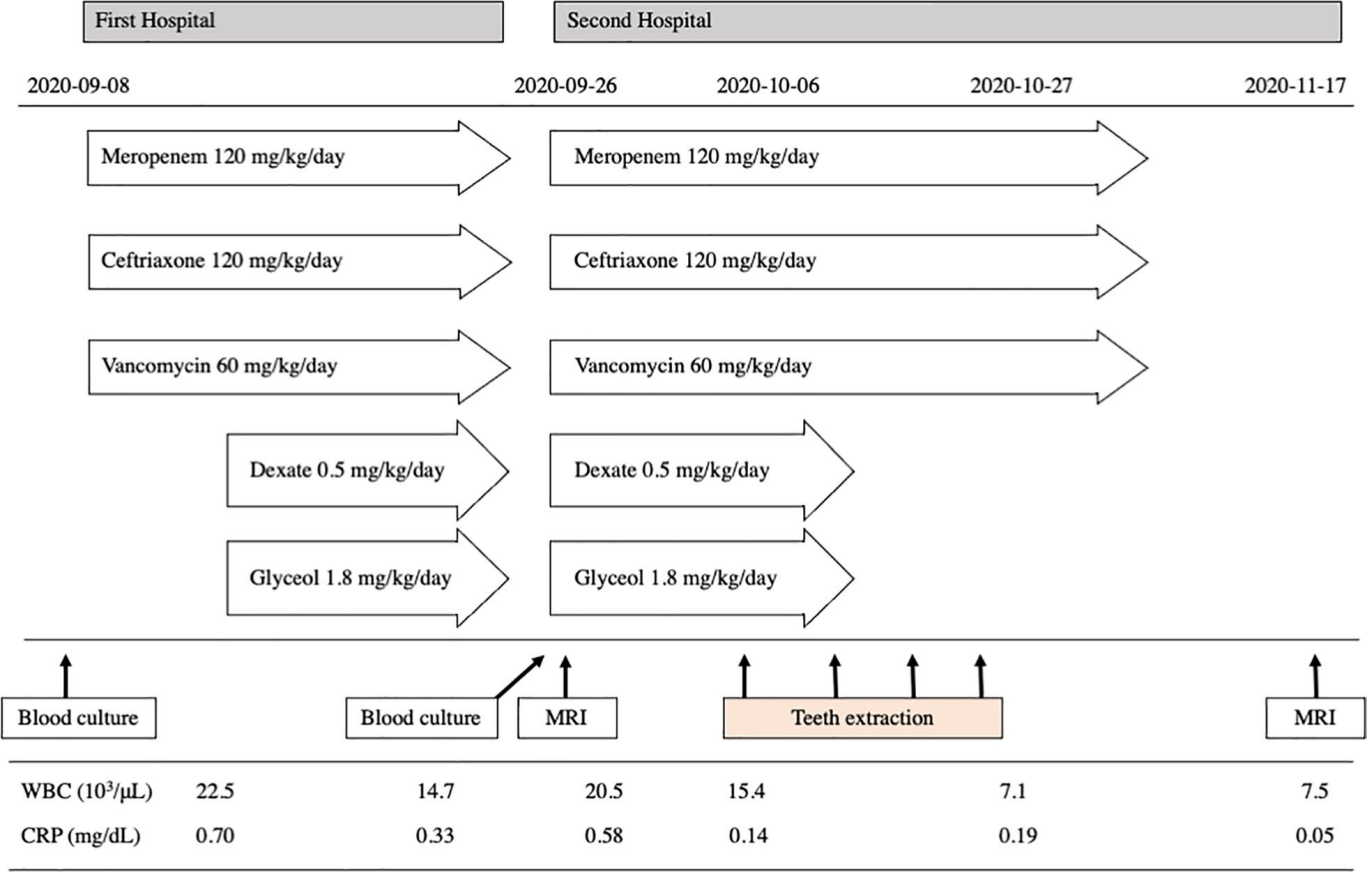
Course of medication and blood data

Our patient was a 7-year and 8-month-old boy (height: 122.1 cm, weight: 18.1 kg) with a complex congenital heart disease diagnosed as complete atrioventricular septal defect, pulmonary atresia, major aortopulmonary collateral artery, and total anomalous pulmonary venous connection with pulmonary vein stenosis. On September 8, 2020, the patient visited a general hospital (the First Hospital) due to fever and headaches. After establishing a diagnosis of multiple brain abscesses, treatment with meropenem (120 mg per kg/day), ceftriaxone (120 mg per kg/day), and vancomycin (60 mg per kg/day) was started (Fig.  1). Blood cultures were obtained. *Streptococcus intermedius* and *Staphylococcus aureus* were detected. Two weeks later, the patient showed improvement in symptoms, such as fever and headache.

**Fig. 2:**
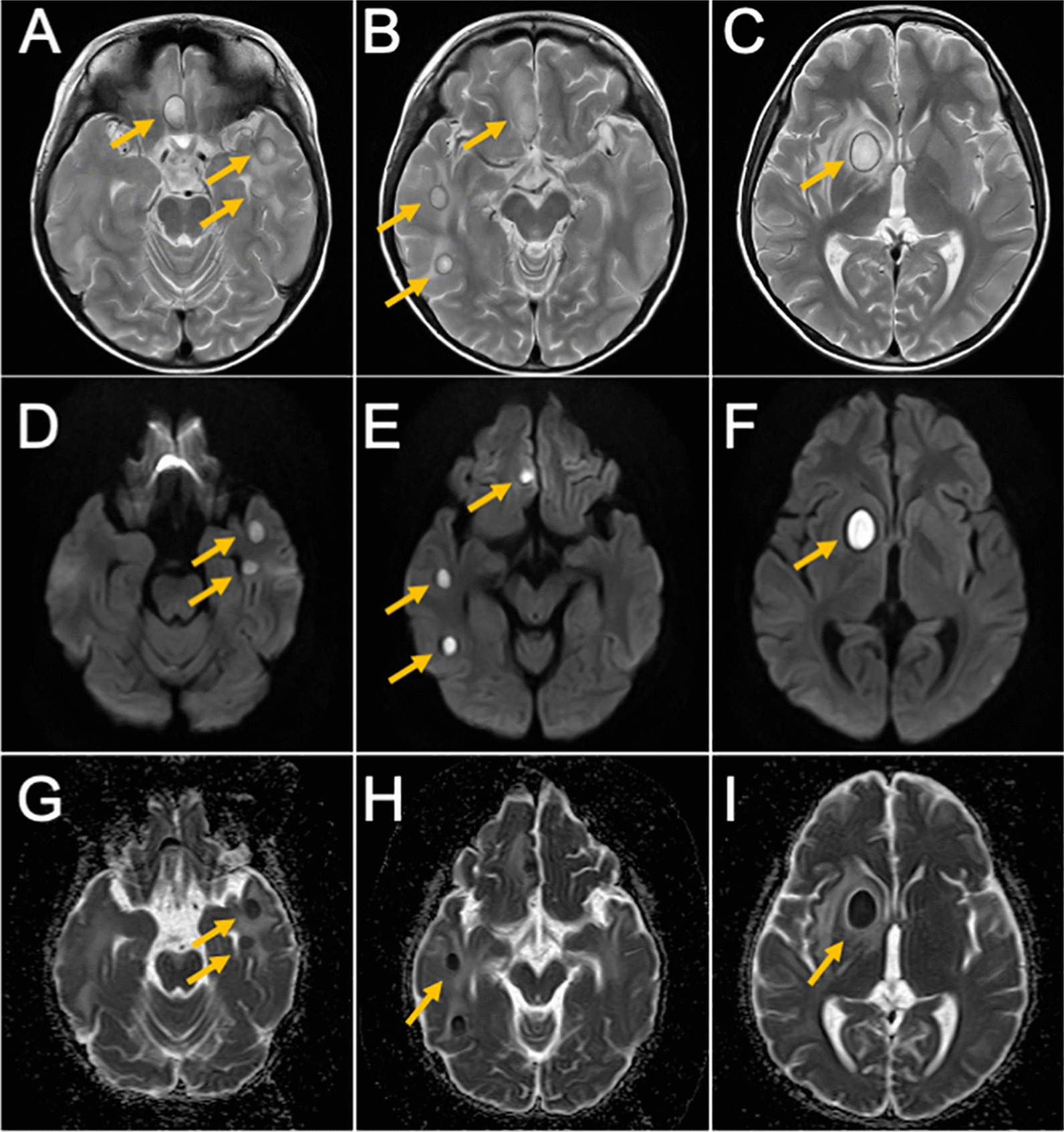
MRI obtained on September 26, 2020. T2-weighted image (**A**, **B**, and **C**), diffusion-weighted image (**D**, **E**, and **F**), ADC map (**G**, **H**, and **I**). Yellow arrows indicate brain abscesses. There were multiple nodular lesions showing ring-like hyperintensity in T2-weighted images and internal hyperintensity and decreased ADC in diffusion-weighted images

However, on September 26, neurological symptoms, such as headache and numbness of the hands and feet, reappeared, for which the patient was transferred to the Second Hospital, to seek further examination and medical treatment due to exacerbation of his brain abscess and circumferential edema on magnetic resonance imaging (MRI). Meropenem (120 mg per kg/day), ceftriaxone (120 mg per kg/day), and vancomycin (60 mg per kg/day) were then started as continuation treatment (Fig.  1), followed by whole-body scanning for the identification of the fungus and source of the infection at the Second Hospital. Accordingly, MRI showed several nodular lesions in the right frontal lobe, right basal ganglia, right thalamus, and bilateral temporal lobes subcortically, with hyperintensity on the rings on T2-weighted images and hyperintensity and reduced apparent diffusion coefficient internally on diffusion-weighted images (Fig.  2). Based on MRI findings, we suspected a hematogenous infection due to multiple brain abscesses. Echocardiography showed no vegetation in the heart, whereas rereading of the positron emission tomography-computed tomography images taken at the previous hospital did not reveal any obvious source of infection in the systemic or otolaryngological areas. Blood culture were obtained and later found negative.

**Fig. 3:**
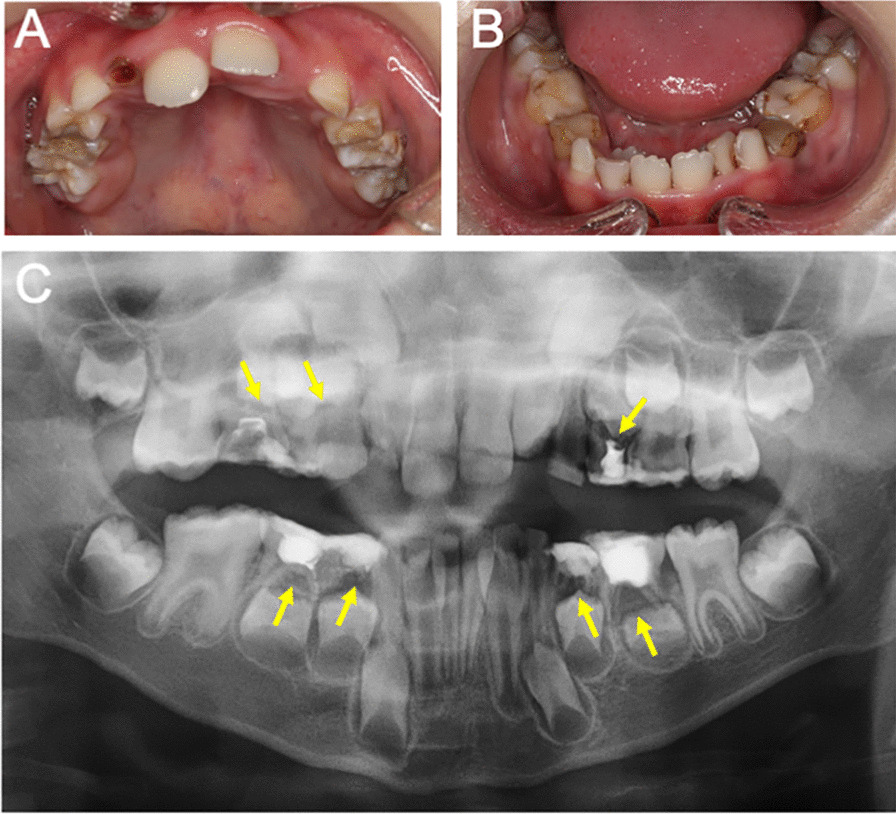
Clinical and radiographic findings of the patient at the initial visit. Intraoral photograph (**A** and **B**) and panoramic radiograph (**C**). Yellow arrows indicate apical periodontitis

**Fig. 4:**
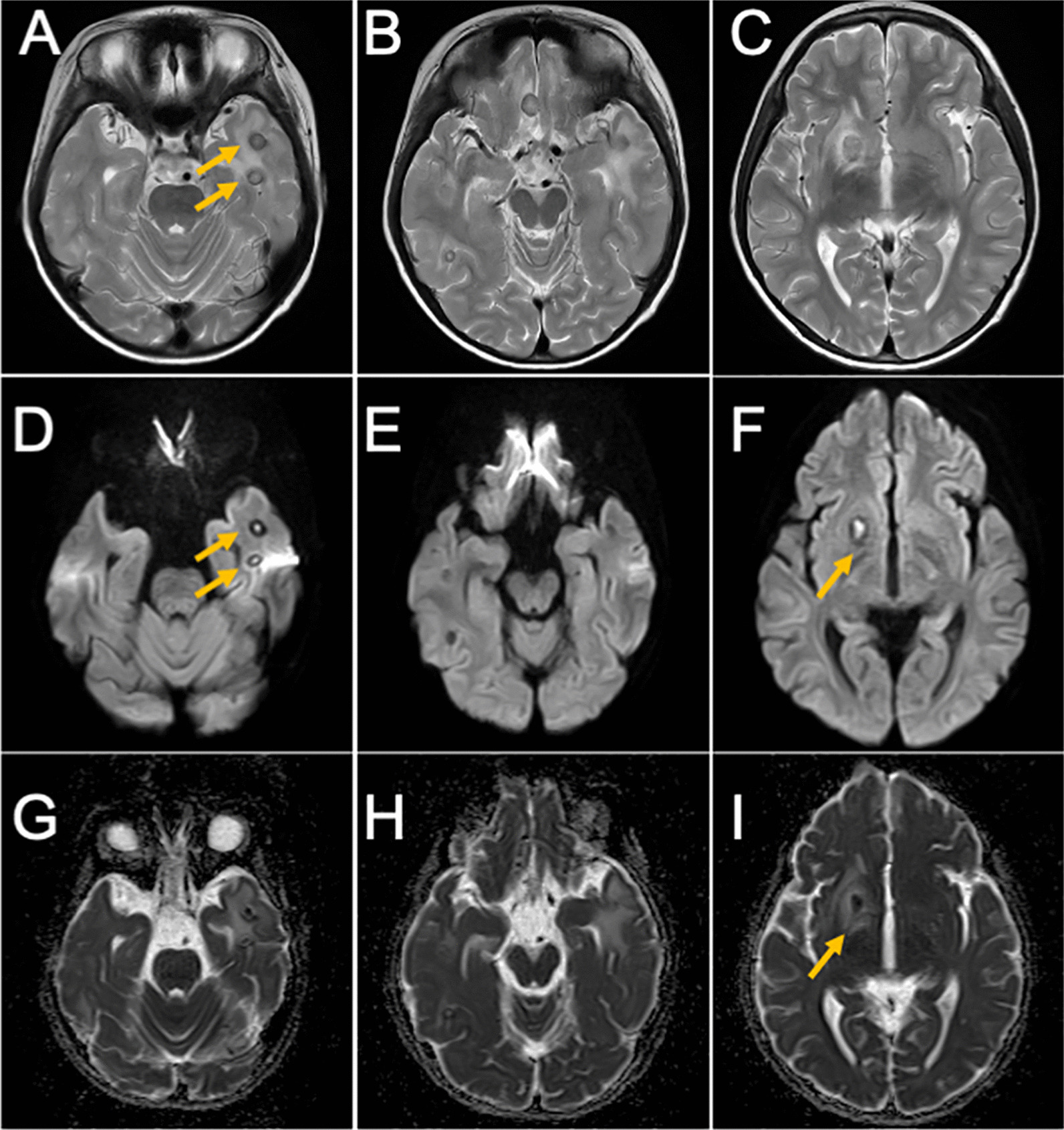
MRI obtained on November 11, 2020. T2-weighted image (**A**, **B**, and **C**), diffusion-weighted image (**D**, **E**, and **F**), ADC map (**G**, **H**, and **I**). Yellow arrows indicate brain abscesses

On September 29, the patient was referred to the Pediatric Dentistry of the Second Hospital for further examination of the source of infection in the oral region. Intraoral views showed unfavorable fillings in the maxillary right deciduous incisor remnant root, mandibular bilateral deciduous incisor late remnant, and maxillary and mandibular bilateral deciduous molars (Fig.  3A, B). Panoramic radiography showed periapical transmission and root resorption in both maxillary and mandibular deciduous molars (Fig.  3C). The causative organism of the hematogenous brain abscess was thought to have depended on the infection of the primary lesion. Examination for the causative factor at our hospital revealed no possible source other than dental infection. Therefore, apical periodontitis of deciduous teeth was considered the source of multiple brain abscesses in this case. All deciduous teeth, including those with infected lesions, were extracted under local anesthesia. The neurological symptoms and headache disappeared immediately after the extraction in late October. Although the administration of the antimicrobial agent was also completed by early November, the patient’s general condition improved considerably afterward. A head MRI performed 1 month after the extraction of the deciduous teeth showed that the brain abscess remained but was noticeably reduced (Fig.  4). The patient was discharged from the hospital on November 17, 2020, given the lack of residual disease activity and the curative course of the patient’s condition.

## Discussion and conclusion

Studies have shown that in cyanotic congenital heart diseases with right to left shunt, such as tetralogy of Fallot or functional single ventricle, venous blood may enter directly into the systemic circulation, thereby increasing the risk for developing brain abscesses [6, 7]. Given that the patient had a cyanotic congenital heart disease with right to left shunt, we speculated that the patient developed multiple brain abscesses caused by apical periodontitis of deciduous teeth. Clinical manifestations of brain abscesses include intracranial hypertensive symptoms, such as headache, vomiting, and disturbed consciousness, as well as neurologic symptoms such as focal seizures, motor paralysis, sensory paralysis, aphasia, and cerebellar ataxia, depending on the location of the abscess [8]. The cornerstones of brain abscess treatment include antimicrobial administration, neurosurgical drainage, and treatment of the infected primary lesion. The treatment of the infected primary lesion is particularly important. Moreover, it is necessary to suspect persistent bacterial infection from the infected primary lesion when neurological symptoms, such as severe headache and nausea, persist despite having administered antimicrobial agents [9, 10].

Approximately 50–80% of cerebral abscesses are reported to be caused by *Streptococcus intermedius,* a gram-positive *Streptococcus* species that reside in the oral cavity [11]. In our patient, *Streptococcus intermedius* was indeed detected in the first blood culture performed immediately after admission. On the other hand, examination by the previous dentist initially determined that there was no need for dental treatment, which prompted continuation of long-term antibacterial agent administration while searching for other causes. In the blood culture obtained 3 weeks after the immediate administration of antimicrobials, *Streptococcus intermedius* was not detected. The reason might be that the antimicrobial therapy was effective. However, the patient’s headache aggravated, his brain abscess increased, and his circumferential edema recurred. Many diseases originating from dental infections can be treated with appropriate treatment. In the current case, removal of the deciduous teeth, which were suspected to have been the source of the infection, seemed to be effective even with serious brain abscess considering that the patient’s symptoms improved after the extraction.

We herein detail our experience with a patient who presented with multiple brain abscesses with primary infection of the apical periodontitis of deciduous teeth. The lack of early causative factor elimination seemed to have aggravated the patient’s brain abscess over a long period. In particular, patients with severe cyanotic congenital heart disease may develop serious brain abscesses, indicating the importance of continued administration of antibiotics during dental treatment, appropriate follow-up after dental treatment, and employment of proper measures when signs of foci formation are observed.

## Data Availability

The datasets used or analyzed during the current study are available from the corresponding author upon reasonable request.

## Abbreviations

MRI: Magnetic resonance imaging
ADC: Apparent diffusion coefficient
WBC: White blood cell
CRP: C-reactive protein
